# Effects of a Cognitive-Functional Intervention Method on Improving Executive Function and Self-Directed Learning in School-Aged Children with Attention Deficit Hyperactivity Disorder: A Single-Subject Design Study

**DOI:** 10.1155/2020/1250801

**Published:** 2020-07-10

**Authors:** Mi Ji Kim, Hae Yean Park, Eun-Young Yoo, Jung-Ran Kim

**Affiliations:** ^1^BODA Visual Perception & Cognition Educational Institution, Republic of Korea; ^2^Department of Occupational Therapy, Yonsei University, Republic of Korea; ^3^College of Human Service, Department of Dementia Prevention and Rehabilitation, Catholic Kwandong University, Republic of Korea

## Abstract

**Background:**

School-aged children with attention deficit hyperactivity disorder (ADHD) face many difficulties with self-directed learning because of their poor executive function. This leads to secondary problems such as learning disabilities and depression, so the role of intervention to improve executive function in school-aged children with ADHD is important.

**Objective:**

The present study is aimed to investigate how cognitive-functional (Cog-Fun) intervention affected executive function of school-aged children with ADHD and the sustainability of these effects. To investigate the effects of changes in the executive function of school-aged children with ADHD through Cog-Fun intervention in self-directed learning.

**Method:**

A single-subject A-B-A research design was employed in this study. Three children aged 9-10 years who were diagnosed with ADHD were selected. A total of 17 experimental sessions were conducted. The Cog-Fun intervention program was implemented during the intervention phase. To measure dependent variables, Behavior Rating Inventory of Executive Function (BRIEF) and Homework Problems Checklist (HPC) were used. Significant changes in executive function assessed by the Children's Color Trails Test (CCTT) and Stroop test were analyzed through two-standard deviation band analysis. Additionally, video clips of task performance were analyzed to examine qualitative performance changes in self-directed learning.

**Result:**

All three participants presented statistically significant changes with a number of near-misses of CCTT and color words score of Stroop test during the intervention. *T*-scores of the Global Executive Composite (GEC) decreased after the intervention, indicating improvement in executive function. The follow-up period revealed retention of the improved executive function. Additionally, self-directed learning improved in all participants after the implementation Cog-Fun intervention.

**Conclusion:**

The study supports the effectiveness of Cog-Fun intervention in improving executive function in school-aged children with ADHD and confirmed that the improvement of executive function ultimately leads to the improvement of self-directed learning performance.

## 1. Introduction

Attention deficit hyperactivity disorder (ADHD) is the most common neurodevelopmental socio-behavioral cognitive disorder in school-aged children; approximately 9–11% of elementary school children have a tendency to develop ADHD [1, 2]. The main problems associated with ADHD are hyperactivity, inattention, and impulsiveness. Repeated failures and experiences of frustration due to these effects result in increased risk of a broad range of mental disorders such as mood, anxiety, eating, and personality disorders [3, 4]. Therefore, it is important to apply appropriate interventions to address the main problems of ADHD.

The behavioral characteristics of ADHD described above have a clear relationship with defects in executive function [5–8]. Executive function is a neuropsychological process that involves behavioral self-control and allows for effective setting, planning, execution, and achievement of goals [9]. Executive function includes inhibition of impulses, shifting between tasks, working memory, planning, and organizational abilities [10]. Defective executive function affects aspects of occupational function, especially academic development of school-aged children since demands for academic autonomy and independence increase at this time [11–13].

School-aged children with ADHD are characterized by low task performance rates and chronic academic challenges, including in self-directed learning, due to defects in executive function [14–16]. For example, these children are not able to write down tasks and related information provided by the school, submit delayed or incomplete homework, and are not able to focus during task performance [14]. This demonstrates one aspect of the difficulties with self-directed learning, which is the ability to control intrinsic processes, with the understanding of the surrounding environment and with their learning behavior to enhance self-knowledge, skills, sense of accomplishment, or personal development through effort. Therefore, interventions that aim at improving executive function are urgently required [12].

A variety of pharmacological and nonpharmacological interventions are available for ADHD. Although there is methylphenidate as a typical drug treatment, it is that drug treatment has a temporary effect and side effects, and the underlying problem cannot be solved. Nonpharmacological interventions include behavioral interventions, neuro-feedback, cognitive training, and restricted elimination diet [17]. Occupational therapy interventions for children with ADHD mainly focused on play, sensory, motor, and cognitive skills [18]. However, interventions taking into account the individual characteristics of school-aged children with ADHD and the interventions related to the process of behavior change and the sustainability of the change were limited.

The cognitive-functional (Cog-Fun) intervention method is an occupational therapy intervention designed to enhance the executive functions of children with ADHD. It aims to aid participants in acquiring strategies for the execution of daily living activities and increase participation in important occupations, to enhance the quality of life of children and their families. Specifically, Cog-Fun interventions were designed based on the Person-Environment-Occupation Model (PEO Model) and the Model of Human Occupation (MOHO) and focused on the executive functions of children with ADHD [19, 20]. Their main properties include setting child-centered goals, providing training in the three execution strategies of “stop, plan, and review,” and aiding children in becoming able to set their own strategies [16].

A previous study that investigated the effects of Cog-Fun intervention in children with ADHD aged between 7 and 9 years showed significant improvements in executive function, occupational performance results, and performance of targeted behaviors [21]. In addition, Maeir et al. conducted a controlled study with 19 children aged between 5 and 7 years old and reported significant improvement in executive function along with improvements in occupational performance and satisfaction, emphasizing the importance of the parents' role in persistently transferring the strategies learned in interventional situations to the home environment [16]. A recent randomized controlled study with 107 children also confirmed improvements in occupational performance and satisfaction [22].

However, there are no studies on school-aged children which have targeted occupational performance in learning. Since there is only one before- and after-intervention study, it is difficult to acquire information on changes in children's functions, other than executive function. Therefore, this study implemented individual experimental methods to generate information related to the behavioral change process and the sustainability of these changes resulting from interventions that consider individual characteristics of children with ADHD; it also investigated the effects of Cog-Fun on the improvement of executive function of children with ADHD. In addition, each case was observed and analyzed to determine the actual changes in self-directed learning performance after change in executive function.

The detailed objectives of this study were as follows:

1. To investigate the effects of Cog-Fun intervention on the executive function of school-aged children with ADHD and the sustainability of these effects
2. To investigate the effects of changes in the executive function of school-aged children with ADHD through Cog-Fun intervention in self-directed learning

## 2. Materials and Methods

### 2.1. Study Design

This study employed an A-B-A design, which applied a withdrawal scheme in single-subject research. After a total of 16 sessions twice a week, one follow-up session after two weeks was conducted to determine the sustainability of the effects of intervention.

### 2.2. Participants

This study was conducted with children diagnosed with ADHD and lived either in Seoul or Gyeong-gi Province. The inclusion criteria were (1) diagnosis of ADHD by a medical doctor or clinical psychologist, (2) attending school from 3rd to 6th grade, (3) score of 14 or higher in Conners Abbreviated Rating Scale (CARS), (4) score of 55 or higher in social quotient on the Social Maturity Scale and at a level where education is possible, (5) reported difficulties in performing homework, (6) no overlapping visual, auditory, or physical disability, and (7) parental agreement to the study.

### 2.3. Assessment Procedure

The experiment combined a measurement of executive function and self-directed learning performance; Behavior Rating Inventory of Executive Function (BRIEF) and Homework Problem Checklist (HPC) were conducted at baseline, after the intervention session, and during the follow-up session to measure changes in overall function. In addition, in each session, the Children's Color Trails Test (CCTT) and Stroop test for children were conducted, and performance in self-directed tasks was video-recorded.

BRIEF is used to measure executive function in school-aged children between 5 and 18 years old and includes the following items: inhibition, shifting tasks, emotional control, initiation, working memory, planning/organization, organization of materials, and monitoring [23]. Test-retest reliability of parent assessment is 0.72-0.84.

HPC is a parent-reported assessment scale composed of 20 items used to identify the level of difficulty in the performance of homework [24]. This tool has a high internal consistency of 0.90-0.92 and is structured in a 4-point Likert scale where “0” indicates “not at all” and “3” “very frequently”. Homework performance was chosen as a self-directed learning activity, and, through HPC, the changes in homework performance before and after the intervention were investigated.

CCTT measures executive function, mainly focusing on attention, cognitive flexibility, and susceptibility to interference [25, 26]. This study used the standardized Korean version of the Color Trails Test. To prevent a ceiling effect in the test, a random number generator application was used to randomly designate each number to a location, and different test sheets were used for each session.

The Stroop Color and Word Test Children's Version is used to measure cognitive flexibility, response inhibition, attention, automation, reading, semantic memory, and self-control [26]. This study used a color-word task that identifies executive function such as cognitive flexibility and response inhibition of children with ADHD. The test-retest reliability of the task is 0.73 [27].

Lastly, to assess satisfaction with the program, Treatment Evaluation Inventory-Short Form (TEI-SF) was employed at the end of the study term. TEI-SF, a short form of TEI, was developed by Kelly et al. in 1989 to evaluate parents' perception with respect to children who received the therapy [28]. This study used 2010 translated version of Kim to identify parental perceptions on the appropriateness, effects, and ethics of the program [29].

### 2.4. Cog-Fun Program

Cog-Fun is an intervention method designed to enhance executive function and self-efficacy based on occupational models [19]. There are two major intervention models for children and adolescents. This study considered both the functional level and age of the participating children when forming the program. The intervention consisted of a total of ten 60-minute sessions, provided twice a week.

Step A, which increases adaptive self-awareness of the children, was provided over two sessions. The initial session evaluated the occupational profile of the child through Child Occupational Self-Assessment (COSA). One goal was set after occupational consultation. The second session consisted of watching video clips related to ADHD. Watching the clips aims at increasing the child's understanding of ADHD and helps them substitute problematic behaviors and realize their own challenges.

Step B, which is designed to develop strategies for executive function improvement, progressed over six sessions together with step C, which modifies and restructures the environment. The child played games related to the themes of each session and cognitive tasks provided by the therapist and was trained in stop, plan, and review strategies by following appropriate protocols [21]. Specialized strategy training sessions were provided to explore and apply their own strategies based on therapeutic learning experiences. While intervention was in progress, a Daily Occupational Goal Planner (DOGP) was provided to children to help them continue to practice the acquired strategies in their daily life. There was consultation with the parents after each session. During consultation, therapists provided parent education that enhances the understanding of children with ADHD and appropriately customizing environment for each child to perform tasks.

The final step D summarized the whole process and integrated prior steps. This step progressed over two sessions focusing on preparative activities for a creative project that outlines their own occupational profile, goals, and acquired strategies.

### 2.5. Data Analysis

This study documented all outcome values of the CCTT and Stroop test for children for each session and presented them using visual graphs. Significant changes in executive function were analyzed through two-standard deviation band analysis. Additionally, video clips of task performance were analyzed to examine qualitative performance changes in self-directed learning. Finally, executive function behavior assessment was conducted and a Homework Problem Checklist was used before and after interventions and during the follow-up sessions to analyze changes in executive function and self-directed learning.

## 3. Results

### 3.1. Participants

Three subjects who met the selection criteria were selected. All three subjects are school-aged male children aged 9 to 10 years who have been diagnosed with ADHD. Participants 1 and 3 were taking methylphenidate, and participant 2 was not receiving medication. As a result of the Social Maturity Scale (SMS) used to measure educational possibilities, all participants were educable or above. Detailed characteristics of participating students are presented in Table 1.

### 3.2. Changes in Executive Function

#### 3.2.1. Executive Function Changes following CCTT

**Figure 1:**
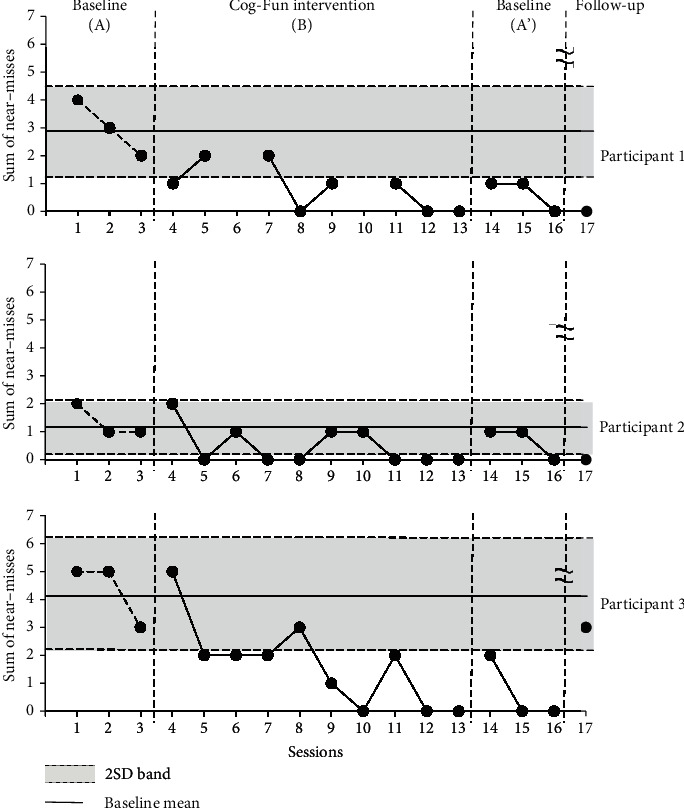
Sum of near-misses in Children's Color Trails Test (CCTT) for executive function during baseline and intervention periods.

After CCTT 1 and 2, the numbers of approximate errors from each trial were summed, and changes were observed as the study progressed. The near-miss index reflects the impulses of the participant, and a low number of near-misses suggest fewer errors due to impulsive behavior. Compared to baseline period A, the number of near-misses during the intervention period revealed mean decreases of 2.3 for participant 1, 0.8 for participant 2, and 2.6 for participant 3. According to the 2SD method (Figure 1), all three participants presented with a number of near-misses during the intervention period outside the 2SD band, confirming that the decrease in the number of near-misses was statistically significant.

#### 3.2.2. Changes in Executive Function following the Stroop Test

**Figure 2:**
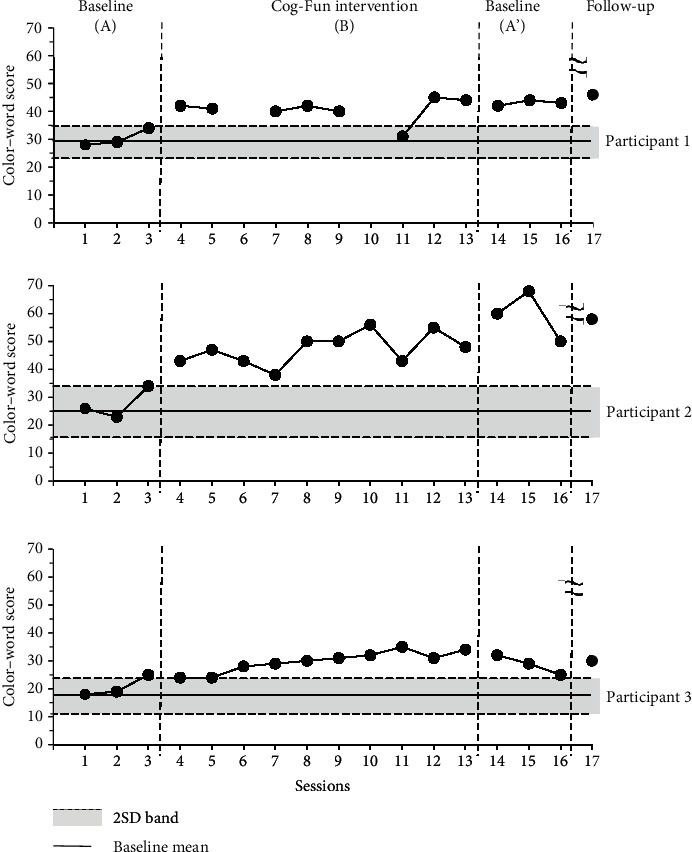
Color-word score in Stroop Color and Word Test Children's Version for executive function during baseline and intervention periods.

This study used the color-word score as a dependent variable, because it informs on the ability of the child to inhibit their impulses and allows for the analysis of changes in scores. A higher score reflects improved cognitive flexibility and response inhibition. The resulting graph revealed that the scores of the three participants fell consistently outside the 2SD band in all intervention periods (Figure 2); therefore, the results were statistically significant. In addition, mean increases in scores during the intervention period were 10.3 for participant 1, 19.33 for participant 2, and 9.2 for participant 3, compared to baseline period A.

#### 3.2.3. Executive Function Changes following BRIEF

The results of BRIEF, which tests the executive function of inhibition, task shifting, emotional control, initiation, planning and organization, organization of materials, and monitoring occupation, indicate an improvement in executive function when *T*-scores and percentiles are decreased. When changes in the Global Executive Composite (GEC) were examined, all three participants showed improved executive function; *T*-scores decreased in the GEC after the intervention from 77 (98%) to 72 (97%) for participant 1; from 74 (97%) to 67 (94%) for participant 2; and from 79 (98%) to 67 (94%) for participant 3. *T*-scores during the follow-up period revealed retention of the improved executive function. When the test results were examined by item, participant 1 presented with the greatest *T*-score changes in task shifting, planning and organization, and inhibition control; participant 2 showed the greatest changes in task shifting, inhibition, and working memory; participant 3 showed the greatest changes in activity initiation, planning and organization, and working memory. The follow-up test confirmed that all three participants maintained the improved executive function without any major changes (Table 2).

### 3.3. Changes in Self-Directed Learning

#### 3.3.1. Changes in Self-Directed Learning by Session Video

In every session, a task performance process consisting of “preparing-performing task-cleaning up” was video recorded and analyzed through a narrative documentation method to determine the actual changes in self-directed learning task performance along with the changes in executive function. Participant 1 was able to achieve the initial goal of “becoming good at doing homework myself” through the Cog-Fun intervention process. Regarding executive function, the participant improved planning and organization and environment structuring; the participant remembered the school schedule and their extracurricular activities, sets an alarm for self-directed learning times, and organized their own space and school materials. Participant 2 is trained with the aim to manage their schedule independently. After intervention, the participant prepared the task, performed it independently, and remembered information related to the schedulewithin everyday routines during the weekends. Participant 3 sets the goal to “do well in homework and finish on time.” The participant generated a daily schedule table as a planning and checking strategy after looking at the time. At home, the participant persistently practiced using a whiteboard with their parents and was later able to perform all activities independently. In addition, as planning for time increased and the task was not completed on time, the participant was less emotionally overexcited. All three participants improved their planning and organization of self-directed learning and showed self-confidence and satisfaction in task performances.

#### 3.3.2. Self-Directed Learning Changes following HPC

**Figure 3:**
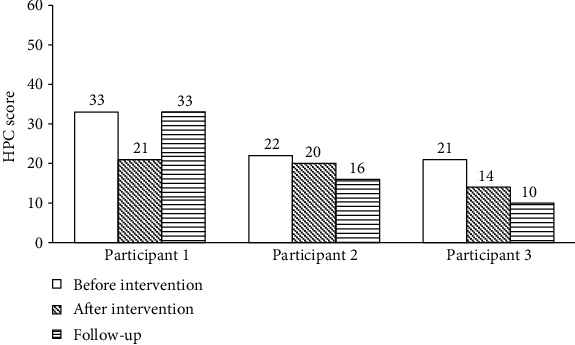
Homework Problem Checklist (HPC) for self-directed learning.

HPC scores, which determine the degree of problematic performance of self-directed learning (homework), become lower when problematic behavior is attenuated. Participant 1 showed a decrease of 12 points after the intervention. The greatest changes were in reminding the participant to do homework, daydreaming or fidgeting with objects while performing homework tasks, and taking a long time to complete homework. Participant 2 showed a 2-point decrease, and the problematic behaviors that improved were reminding the participant to do homework, being easily distracted, and making careless errors. Participant 3 demonstrated changes in daydreaming or fidgeting with objects while performing homework tasks and performing homework task in a messy and unorganized manner. The total score was 14 with a 7-point decrease. Participants 2 and 3 showed a continuous decrease in problematic behaviors during homework performance during the follow-up period, thus maintaining improved self-directed learning (Figure 3).

#### 3.3.3. Program Satisfaction Evaluation

The results of TEI-SF showed that all three parents perceived the Cog-Fun program positively and responded that it improved functional performance with long-lasting effects.

## 4. Discussion

This study investigated the effects of Cog-Fun intervention on executive function and self-directed learning enhancement for school-aged children with ADHD. Changes in executive function were observed through CCTT and Stroop color-word tests at every session, and task performance was analyzed to trace the changes in self-directed learning. In addition, BRIEF and HPC were used to measure and analyze executive function and self-directed learning over pretest, posttest, and follow-up periods. As a result, executive function and self-directed learning of all participants improved after Cog-Fun intervention.

The assessment results per session showed statistically significant changes in the number of near-misses and color-word scores, which are closely related to impulsiveness and inhibition ability of executive function. The results of BRIEF, which was conducted before and after the intervention, showed a mean decrease of -5.06 points (range: -3.90 to -7.20) in the executive function subitem *T*-score, suggesting improved executive function. As for self-directed learning performance, all participants reviewed and became aware of their schedules in narrative record analysis for each session and showed changes in performance such as using a notice board or setting time to independently perform the task. The HPC results before and after the intervention also confirmed improvement in self-directed learning.

The subitems of working memory, planning and organization, and organization of materials in BRIEF showed greater score changes after implementation of Cog-Fun intervention than emotional control or inhibition control. This finding is not consistent with Maeir et al. [30], which reported that changes in impulse inhibition, task shift, and working memory were greater than changes in other subitems of executive function after implementation of Cog-Fun. These differences may result from the fact that the participants of our study set goals to perform tasks while managing time by themselves and focused on intervention strategies of planning and organization and organization of materials. In relation to self-directed learning, the greatest changes were in “decreasing the time taken to do homework,” “remembering to take the homework to school,” and “fewer errors from hurrying and carelessness” in HPC, in concordance with BRIEF.

Moreover, when the score changes between pre- and posttest were examined, there was a mean decrease of 3.90 points for participant 1, 4.10 points for participant 2, and 7.20 points for participant 3, indicating improved executive function. These values and the degree of changes are concordant with results from previous studies that implemented Cog-Fun [16, 21, 22, 30]. Executive function improvement through Cog-Fun intervention is related to the integrated mechanism of various factors associated with the intervention principles. Parent-related factors are (1) setting realistic goals, (2) learning execution strategies in home environments and integrating them into everyday routines, and (3) supporting use of execution strategies when the child is training and modifying the environment as needed. Child-related factors are (1) effective acquisition of execution strategies, (2) improvement of performance skills, and (3) increasing self-efficacy [22]. During the study period, parents were educated on the characteristics of ADHD and how to objectively understand their child's abilities. They were provided with clear, accurate, and appropriate feedback and implemented environment modifications to allow the child to achieve successful occupational performance. Consequently, as sessions progressed, the children eagerly stated “I can do better now” and “I took a picture of what I have done,” thus showing self-efficacy. This finding reflects their increased self-perception through accumulated experiences of success after Cog-Fun intervention and after encouraging children to challenge themselves with new tasks to achieve their goals.

As such, the results of this study have similarities and differences from those of previous studies that implemented Cog-Fun intervention program. Because the execution function includes various aspects such as behavioral regulation and metacognitive aspects [10], this can lead to a variety of results depending on the target and goal setting. This study differs from the previous study in that it deals with learning-related problems faced by school-aged children with ADHD [21, 22, 30]. In addition, through the application of a single subject study design, it has a clinical significance in that this study generated information related to the children's executive function change process and presented the practical effect of self-directed learning performance after change in executive function.

There are some limitations in this study. The first one is the Hawthorne effect; despite emphasizing parent education and involvement in interventions, according to Cog-Fun intervention protocol and characteristics, BRIEF and HPC showed a tendency for parental reporting. These tools were selected in consideration of prior Cog-Fun study designs, but the Hawthorne effect could not be neglected as most studies used parent report assessment tools for measuring dependent variables. The second limitation is that not enough measures were taken to ensure objective analysis of participant performance. When studying the effects of interventions, such as Cog-Fun, that enforce the participant to select and develop their own strategies to solve problems, it may be helpful to use the Goal Attainment Scale (GAS), which measures performance changes of specific goals that are difficult to be identified with standardized assessment tools [31]. Also, a more objective analysis may have resulted if several therapists interpreted the video recording or a triangulation method was used.

Based on this study, prospective studies may be performed to investigate the effects of Cog-Fun by separating different characteristics of ADHD, such as attention deficit dominant type, hyperactivity impulse dominant type, and complex type. The age range of participants may include preschool-age children to high school students to investigate whether the interventions are effective for children and adolescents with ADHD.

## 5. Conclusion

This study investigates the effects of Cog-Fun intervention on executive function and self-directed learning in school-aged children with ADHD. After Cog-Fun intervention, executive function improved in all participants. All participants showed changes in working memory, planning and organization, and organization of materials. In addition, the enhanced executive function was maintained after termination of the intervention. Self-directed learning also improved for all participants after Cog-Fun intervention. As the intervention progressed, the children generated their own planning tables, and an effort to enhance working memories by modifying performance strategies was observed. Ultimately, the Cog-Fun intervention method improved executive function and self-directed learning performances in children with ADHD.

## Figures and Tables

**Table 1:** Demographic characteristics of participants.

Characteristics		Participant 1	Participant 2	Participant 3
Age		9 y 8 m	10 y 3 m	9 y 4 m
Sex		Male	Male	Male
Diagnosis		ADHD	ADHD	ADHD
Drugs		Methylphenidate	No medication	Methylphenidate
CARS-P		24	18	28
SMS	Social age	8.3	13.4	9.5
	Social quotient	85	130	102

CARS-P: Conners Abbreviated Rating Scale-Parents form; SMS: Social Maturity Scale.

**Table 2:** Executive function changes following BRIEF.

*T*-score (%)									
Scale/index	Participant 1			Participant 2			Participant 3		
	Before intervention	After intervention	Follow-up	Before intervention	After intervention	Follow-up	Before intervention	After intervention	Follow-up
Inhibition	69 (96)	62 (91)	66 (94)	78 (98)	69 (96)	62 (91)	78 (98)	69 (96)	66 (94)
Shift	81 (99)	67 (96)	67 (96)	71 (97)	60 (85)	57 (79)	81 (99)	84 (99)	77 (99)
Emotional control	76 (98)	76 (98)	62 (90)	71 (98)	67 (92)	56 (73)	78 (99)	71 (98)	73 (98)

*Behavioral regulation index*	78 (98)	71 (96)	67 (95)	77 (98)	68 (95)	60 (84)	83 (99)	77 (98)	75 (97)

Initiate	66 (93)	72 (98)	69 (98)	68 (98)	63 (88)	47 (49)	72 (98)	56 (79)	56 (79)
Working memory	76 (98)	72 (97)	74 (98)	67 (95)	60 (85)	58 (81)	69 (96)	56 (77)	58 (81)
Plan/organize	73 (98)	69 (94)	71 (97)	67 (93)	63 (90)	54 (70)	67 (93)	52 (66)	52 (66)
Organization of materials	55 (74)	45 (35)	52 (63)	55 (74)	58 (78)	45 (35)	58 (78)	49 (45)	39 (18)
Monitor	75 (99)	69 (98)	62 (91)	72 (98)	69 (98)	56 (79)	78 (99)	72 (98)	72 (98)

*Metacognition index*	74 (98)	69 (96)	77 (99)	69 (96)	65 (91)	53 (64)	73 (98)	58 (77)	57 (73)

GEC	77 (98)	72 (97)	74 (97)	74 (97)	67 (94)	56 (77)	79 (98)	67 (94)	65 (92)

Values are presented as score (%); GEC: Global Executive Composite.

## Data Availability

The data used to support the findings of this study are available from the corresponding author upon request.
